# A realist review of family-based interventions for children of substance abusing parents

**DOI:** 10.1186/s13643-015-0158-4

**Published:** 2015-12-18

**Authors:** Amelia M. Usher, Kelly E. McShane, Candice Dwyer

**Affiliations:** Ryerson University, 350 Victoria St., Toronto, ON M5B 2K3 Canada

**Keywords:** Addiction, Substance abuse, Children, Family, Parent, COSAP, Program evaluation, Realist review/synthesis, Intervention

## Abstract

**Background:**

Millions of children across North America and Europe live in families with alcohol or drug abusing parents. These children are at risk for a number of negative social, emotional and developmental outcomes, including an increased likelihood of developing a substance use disorder later in life. Family-based intervention programs for children with substance abusing parents can yield positive outcomes. This study is a realist review of evaluations of family-based interventions aimed at improving psychosocial outcomes for children of substance abusing parents (COSAPs). The primary objectives were to uncover patterns of contextual factors and mechanisms that generate program outcomes, and advance program theory in this field.

**Methods:**

Realist review methodology was chosen as the most appropriate method of systematic review because it is a theory-driven approach that seeks to explore mechanisms underlying program effectiveness (or lack thereof). A systematic and comprehensive search of academic and grey literature uncovered 32 documents spanning 7 different intervention programs. Data was extracted from the included documents using abstraction templates designed to code for contexts, mechanisms and outcomes of each program. Two candidate program theories of family addiction were used to guide data analysis: the family disease model and the family prevention model. Data analysis was undertaken by a research team using an iterative process of comparison and checking with original documents to determine patterns within the data.

**Results:**

Programs originating in both the family disease model and the family prevention model were uncovered, along with hybrid programs that successfully included components from each candidate program theory. Four demi-regularities were found to account for the effectiveness of programs included in this review: (1) opportunities for positive parent-child interactions, (2) supportive peer-to-peer relationships, (3) the power of knowledge, and (4) engaging hard to reach families using strategies that are responsive to socio-economic needs and matching services to client lived experience.

**Conclusions:**

This review yielded new findings that had not otherwise been explored in COSAP program research and are discussed in order to help expand program theory. Implications for practice and evaluation are further discussed.

**Electronic supplementary material:**

The online version of this article (doi:10.1186/s13643-015-0158-4) contains supplementary material, which is available to authorized users.

## Background

Substance abuse is a persistent and longstanding issue in society. It is estimated that 7 to 11 % of adults have an alcohol use disorder in the USA and UK, respectively [1], and in Canada the prevalence of adult substance abuse (including both drugs and alcohol) is estimated to be 11 % [2]. The impact of drug and alcohol abuse extends beyond the individual user to other family members, and in particular children. It is estimated that approximately 1 in 4 children in the USA are exposed to alcohol abuse in their family [3] and that between 8 and 11 million American children are living with an alcohol or drug user in the home [4, 5]. Similar rates have been noted in the UK and elsewhere [6]. Children of substance abusing parents (COSAPs) are at increased risk of negative psychosocial and developmental outcomes such as depression, anxiety, social isolation, conduct and behavioural problems and lower academic achievement [7–9].

Over the past few decades, a small number of programs specifically targeting COSAPs have emerged and many take a family-focused approach to intervention. Family-based programming seeks to elicit change by involving multiple family members in an effort to strengthen family functioning and reduce negative childhood outcomes. It is argued that family-based intervention programs can be more effective than working with children or parents alone [10–12]. Evidence suggests that these programs can yield positive changes for COSAPs (e.g. [13]); however, it is unknown how or why these programs achieve outcomes and an attempt at program theory has yet to be articulated.

### Objectives

A systematic review of COSAP programs conducted in 2012 examined effect sizes of nine programs and concluded that there was preliminary evidence for their effectiveness, particularly for proximal outcomes such as family relationships, social behaviours and program-related knowledge [13]. That particular review, however, was not limited solely to family-based programs nor did it delve into what contexts and mechanisms might impact COSAP program outcomes. Indeed, researchers stress that COSAPs are a heterogeneous group [7] and as such will experience different risk factors, likely differentially impacting treatment responsiveness and the pathways through which outcomes are achieved. Further, not all COSAPs will experience significant adversity. At present, it is unknown how and why particular programs are effective and in what contexts are most likely to do so.

The current study sought to fill this gap by adopting a theory-focused approach to improve understanding family-based interventions for COSAPs. This realist review focused on the following key research questions: What patterns of contextual factors and mechanisms can be identified, and how do they generate outcomes? Can a COSAP program theory be articulated and refined, and if so, what are the implications for program implementation? A theory-building approach was used to guide to this realist review.

## Methods

### Rationale for using realist review

This review seeks to synthesize existing knowledge of family-based interventions for COSAPs and articulate a theoretical framework for how such programs work. Realist review was chosen as the methodological approach for this study. A realist review, also known as a realist synthesis, is a type of systematic review that examines research evidence on social interventions in an effort to explain how and why they work, or do not work, in particular contexts [14]. The process of conducting a realist review has been detailed extensively through the Realist and Meta-narrative Evidence Synthesis: Evolving Standards (RAMESES) project [15, 16].

A realist approach to a systematic review is ideal for examining social interventions, particularly those delivered in community settings, because it is recognized that programs are rarely delivered in precisely the same way, nor will they have the same outcomes, due to contextual variables that can never be fully controlled [14, 17]. As such, multiple forms of evidence are included in a realist review, including qualitative research and grey literature, both of which are typically excluded from traditional systematic reviews. Decisions on the merits of document inclusion favour their potential for theoretical contribution over methodological hierarchy of empirical studies [18, 19]. Many COSAP programs are delivered in community settings, which often lack the resources to rigorously evaluate their interventions. Further, while there are published research studies that use experimental designs, many evaluations exist as grey literature, which typically do not reach broader academic audiences.

Part of theory refinement process of a realist review is to examine the relationship between contextual factors and outcomes and the underlying mechanisms that connect the two [14]. This is often referred to as a context-mechanism-outcome (CMO) configuration. Mechanisms can be thought of as underlying processes that operate in certain contexts to generate outcomes. Mechanisms are not visible; rather they are inferred from observable data, are context dependent and generate outcomes [17]. Contexts, mechanisms and outcomes are extracted during the realist review process and can be thought of as the “data” that provide evidence to support, reject or refine a program theory. CMO configurations are then compiled in order to map patterns of demi-regularities. A demi-regularity refers to a semi-predictable pattern of program functioning, which helps to explicate program theory [20].

This realist review followed practice guidelines and current publication standards for document selection, appraisal, data extraction and presentation of results, as outlined by the RAMESES project [19, 21]. This review does not contain a PROSPERO registration.

### Scoping the literature for candidate program theories

A scoping search was conducted in order to identify any existing theories on family-based addiction interventions with a focus on children, as well as identify key programs or authors of note. We began our scoping with an extensive review of the literature on family-based theories of addiction. This process was guided by the collective professional knowledge of the field and lived experience of family addiction of the research team, as well as consultation with expert informants in the field. Based on this scoping search, we selected two candidate program theories of how parental addiction progressively impacts children, upon which COSAP programs are theoretically based: (1) the family disease model and (2) the family prevention model. These theoretical models of how addiction progresses within the family were helpful for the research team in making preliminary classifications of each program according to underlying addiction theory.

#### Family disease models

The origins of family disease model programs are rooted in the abstinence and 12-step facilitation movements. Addiction is viewed as a family disease, whereby the entire family is affected by one person’s addiction [22]. This theory posits that parental addiction leads to secrecy, shame, codependency and isolation, which in turn leads to child and family dysfunction [6, 22, 23]. Children living in this environment, therefore, are in need of specific intervention within a family context in order to disrupt the cycle of addiction. Consequently, interventions that espouse this philosophy will attempt to break down existing patterns of secrecy and isolation, often by providing education and knowledge to family members about the impacts that addiction has on children [24].

#### Family prevention models

In contrast, the family prevention model views addiction as one of many risk factors that characterize dysfunctional families. This theory suggests that parental addiction leads to poor parenting skills, poor emotion regulation and poor family cohesion, which in turn leads to childhood psychosocial problems, delinquency and eventually substance use [25, 26]. Improving the family environment is key to reducing risk factors for COSAPs [27]. Interventions that adhere to the family prevention model will target risk and resiliency factors such as strong family bonds, supportive parental monitoring and supervision and relapse prevention and substance refusal skills [11, 28].

Both candidate program theories are similar in that they share short-term goals of improving child psychosocial outcomes, parenting behaviours and family functioning, as well as a longer term goal of eventually reducing probability that COSAPs will develop substance use disorders later in life. Both approaches use the family unit as the vehicle for change. However, differences lie within the pathways to achieving those short- and longer term goals. A distinction is made between knowledge versus skill, as well as parent versus child as the primary target for family change. Programs based in family prevention will emphasize skills over knowledge and primarily target parents, while those taking a family disease perspective typically accentuate knowledge and the child’s experience. In sum, while both models ultimately aspire to similar ends (and may even achieve similar goals), the origins and pathways inherent to each model is slightly different. Our goal was to refine these program models and articulate a COSAP program theory which could account for their success (or lack thereof). We hypothesized that differing mechanisms would be at play for each model of program.

### Search process

The document search process began with a systematic search of academic databases in the psychology, social services and health fields including PsycINFO, Medline, Scopus, CINAHL, Social Work Abstracts and Social Services Abstracts, in September 2013. Search terms included [program OR intervention OR treatment OR therapy] AND [child OR youth OR adolescent OR teenager OR student OR COA OR COSAP] AND [substance OR addiction OR drug OR alcohol] AND [family based OR family skills OR parent training OR parenting skills]. Other search engines such as Google and grey literature databanks were used in an attempt to identify grey literature such as community evaluations, government reports, conference proceedings and other documents not found in academic databases. This was an iterative process, and snowball searching was also undertaken by combing through article reference lists to identify any relevant documents that may have been missed through the initial search process. Additional documents were also revealed through correspondence with COSAP program researchers. Snowball searching continued until December 2014 to ensure that all relevant literature was identified. Only documents written in English were considered for inclusion.

### Selection and appraisal of documents

Documents were reviewed in stages at the title, abstract and full-text level to determine whether they met inclusion/exclusion criteria. These criteria were as follows: participants were children between the ages of 6 and 18 who have a parent who is a substance user; the parent with the addiction or another caregiver must attend the program with the child and programs are delivered in a group format. A decision was made by the research team to exclude programs geared towards infants and toddlers, as program format and content would likely be fundamentally distinct from those aimed at school-age children and their parents. Any program that was not explicitly family-based (i.e. did not involve both parents and children as participants) was excluded. Appraisal of selected documents were further screened by consensus for relevance and rigour, defined as ability to make a theoretical contribution to the review and trustworthiness of evidence presented in empirical studies [19]. With respect to trustworthiness, we conceptualize this as the degree to which the findings reported in the included documents were deemed credible based on the methodology described. Given the wide variety of included documents (i.e. published articles and unpublished grey literature) and the fact that many evaluations did not report positive desired outcomes, we believe that the body of evidence that supports this realist review and its findings to be suitably rigorous.

### Data extraction and analyses

An iterative process of data extraction was adopted for this study using a team approach. The research team consisted of a senior academic researcher with expertise in realist methodology, a doctoral level graduate student and a volunteer research assistant. The expertise of team members spanned child and clinical psychology, abstinence and harm reduction, marginalized populations, community-based research and personal lived experience with substance abuse. A coding abstraction sheet template, created by the research team, was used with each document in order to extract data relating to program descriptions, contexts, mechanisms and outcomes. Two members of the research team (AU and CD) independently coded all documents. The coding templates were revised and refined in an iterative fashion throughout the data extraction and analysis phase. Completed abstraction sheets were reviewed as a team for consistency, and disagreements were settled through consensus. Documents were then re-coded according to the final agreed upon coding abstraction sheets. Completed abstraction sheets were then reviewed among all research team members and an initial round of discussion took place about potential CMO configurations emerging from the data. CMO configurations were revised based on consensus, and documents were subsequently re-coded to ensure that CMOs had been properly identified. The research team convened on a regular basis to discuss demi-regularities emerging from the data and their degree of fit with candidate program theories. Demi-regularities were identified and labelled based on patterns of CMO configurations. When recurring patterns of program functioning were noted, CMO configurations were sorted and categorized in an attempt to explain the emerging demi-regularities. In cases where CMO configurations did not fully explain the demi-regularities, the research team returned to the data so as to refine the CMOs. Clarification of demi-regularities was then undertaken through a process of modification or generation of new demi-regularities, based on the CMO data. This was done in an iterative process through team discussions in an attempt to fully elicit sound explanatory themes. Document selection, appraisal and extraction for this realist review followed standard guidelines as outlined by the RAMESES project [19, 21].

## Results

### Document characteristics

**Fig. 1:**
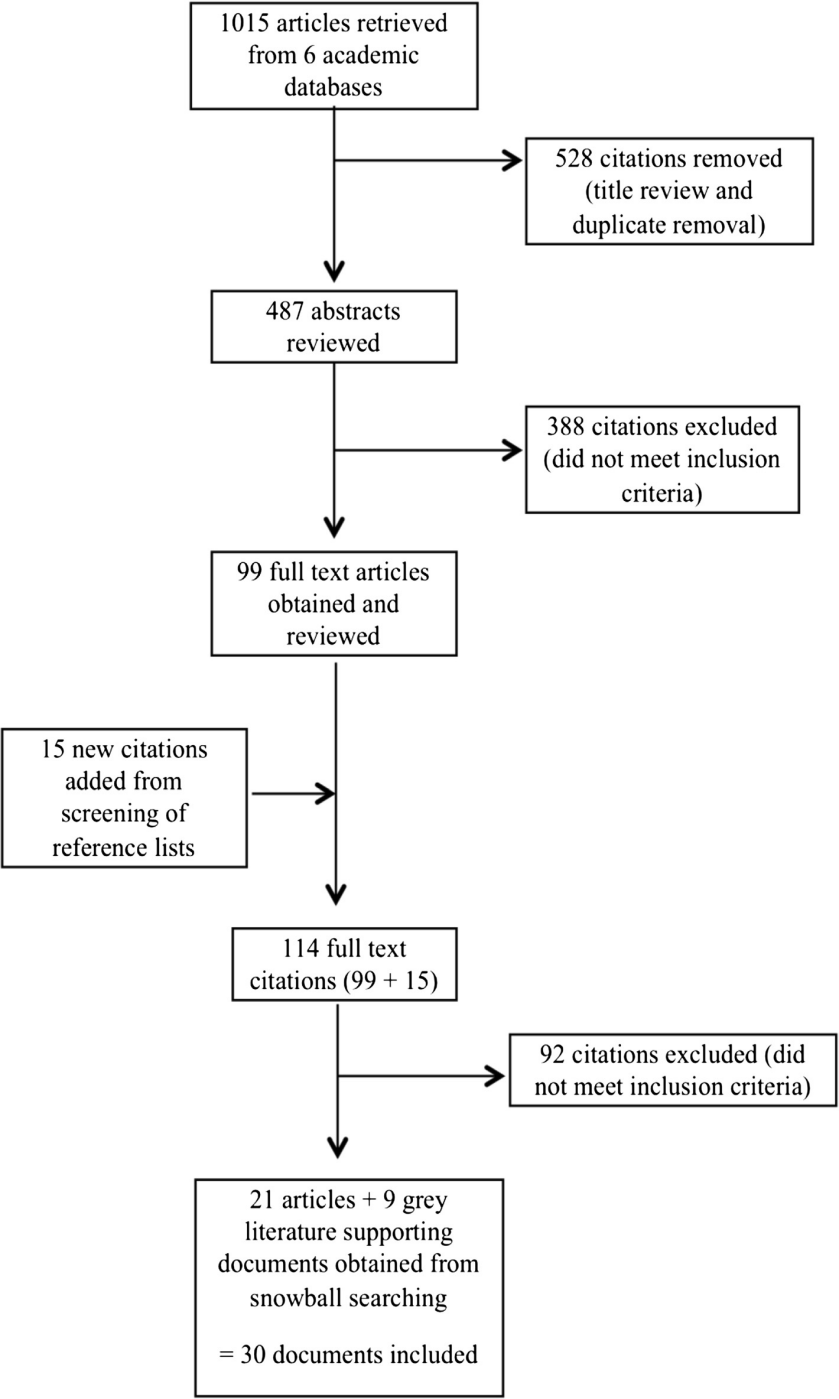
Article search flow chart

Figure 1 presents a flow diagram outlining the document search and appraisal process. Throughout the search process, it became evident that relatively few COSAP programs existed that met the review criteria; however, multiple documents were found as sources of evidence to support each program. A total of 32 documents were retained for this review, spanning 7 different COSAP programs implemented in the USA, UK, Spain and Canada. Types of documents retained included outcome evaluation studies (*n* = 16), grey literature community evaluation reports (*n* = 9), qualitative studies (*n* = 3), book chapters (*n* = 3) and conference presentations (*n* = 1). Additional file 1 outlines the COSAP programs and related documents that were included in this review.

A wide range of outcomes was reported for these programs. The majority were proximal and measured shortly after program completion. We summarized these to reflect general categories: child behavioural changes (e.g. aggression, conduct), child emotional changes (e.g. anxious, depressive, loneliness, self-esteem), parenting (e.g. parenting skills, parent mental health), relapse prevention (reduction in parental substance use) and family cohesion (e.g. bonding, family communication, time spent together). Very few studies were longitudinal and measured reduction or prevention of child substance use later in life. There were also slight variations in terms of dosage, structural format, content, target population (e.g. parents enrolled in concurrent drug treatment, African-American families) and eligibility for program participation (e.g. abstinence during program, demonstrated program commitment, length of time in recovery).

The COSAP programs included in this review were classified by the research team according to their potential support for the two candidate program theories, based on the program descriptions found in supporting documents. For example, programs that promoted a disease-based conceptualization of addiction, emphasized the important of abstinence and implemented a primarily knowledge-based curriculum were categorized within the family disease model. Programs that used a skills-based curriculum aimed at reducing risk factors for substance use and enhancing protective factors within the family were categorized within the family prevention model. Programs that appeared to draw elements from both models were classified as hybrids (see Additional file 1). It is important to note that the purpose of this review was not to rank or compare programs in terms of relative merit or efficacy. Rather, main findings with respect to how and why these programs achieve outcomes are presented below.

### Main findings: demi-regularities

Four demi-regularities were identified in this review as being fundamental in generating positive COSAP program outcomes. These are presented below along with key examples of the contexts, mechanisms and outcomes. Quotations are provided to further illustrate the findings; these represent single examples of multiple instances that were evident in the data.

1. Creating opportunities for positive parent-child interactions

Programs that consistently provided opportunities for positive parent-child interactions were found to produce outcomes of improved family cohesion. Documents from a number of programs (e.g. Safe Haven, Strengthening Families Program (SFP), Moving Parents and Children Together (MPACT), Family Competence Program (FCP)) discussed that the program succeeded in bringing families together for shared time that would not have otherwise been spent. It was frequently noted that providing opportunities for parent-child interactions in an enjoyable and supportive environment (context) led to improvements in family cohesion (outcome).

We identified the mechanism of *hopeful enjoyment* through which this outcome is achieved. Providing multiple opportunities for positive parent-child interactions (context) during the program was found to foster a sense of joy and pleasure (mechanism) among family members and an increased sense of hope that the family unit could be restored (mechanism). A qualitative evaluation of MPACT program provides a useful example of this process:

I think the sheer fact that we went every week and we didn’t miss a week and we all did it together, just that alone I think … It made us feel good about ourselves ([29], p.3).

Our review documented multiple instances of programs that encouraged families to spend time together in a supportive and non-punitive environment. This allowed parents to develop empathy (mechanism) for their children, and in turn children were allowed a safe space (context) to express themselves to their parent during the program [30]. Having parents and children attend together (context) increased positive interactions (outcomes) and encouraged children to feel loved and appreciated by parents (mechanism) [27].

Another useful illustration of this demi-regularity is an example where desired program outcomes were not achieved. The Focus on Families (FOF) evaluation indicated that the program did not achieve desired outcomes for family cohesion [31–33]. In this case, program structure was such that children did not attend all sessions with parents, suggesting a lack of sufficient opportunities for positive parent-child interactions (context). We hypothesize that the mechanism of *hopeful enjoyment* did not have sufficient opportunity to fire in this case. Further, older children actually reported negative effects of parental involvement, suggesting that attempts by parents to increase parent-child interaction time were not only lacking in enjoyment but were in fact met with rejection. The authors of that paper hypothesized that older children who were accustomed to lack of supervision (context) perceived increased family time as an unwelcome intrusion [32]. This provides further evidence that the mechanism of *hopeful enjoyment* needs to be triggered in order for positive outcomes in family cohesion to occur, in the context of child age and prior experience with parental supervision. Programs that facilitate positive parent-child interactions can help families achieve a restored connection when it is developmentally appropriate for them to do so, as is more likely to be the case for younger children. For older children who are at an individuation developmental stage, attempts at eliciting hopeful enjoyment of family interactions may misfire and fail to yield positive outcomes. The accumulation of CMO evidence found in support of this demi-regularity suggests that positive parent-child interactions occurring within a safe and supportive environment, which can be actively fostered by the COSAP program, are instrumental in yielding increased family cohesion.

2. Supportive peer-to-peer relationships

Environments that fostered supportive peer relationships among child participants and among parents were noted across many programs as being instrumental in achieving positive child psychosocial outcomes and to a lesser extent positive parenting outcomes. Evidence was found to support this process in a couple of ways. First, supportive peer-to-peer relationships between the child participants elicited mechanisms of *trust and safety* within the group as well as *validation of experience*. Improvements in child psychosocial functioning were consistently reported in these cases (e.g. [29, 34, 35]). Social isolation (context) is common among children living with parental substance use, and the mere fact of being placed in a supportive group of their peers (context) allowed for feelings of safety (mechanism) to emerge and enabled the sharing of experiences (outcome). For example, a qualitative evaluation of the Betty Ford Children’s Program, which was described as purposefully grouping children according to similar age (context), demonstrates this finding:

I have a lot of, you know, really close friends but they … can’t relate to my situation … you come here and you meet friends who are just like you ([36], p. 389)

Conversely, negative peer-bonding between child participants was noted in one COSAP program evaluation [37], further suggesting that the *trust and safety* mechanism is instrumental in achieving positive child behavioural outcomes. This finding would suggest that attention to participant characteristics such as similar age and lived experience, when forming program peer groups, is warranted as it will facilitate positive peer-bonding and will more likely trigger perceptions of trust and safety.

Second, we noted that parent participants who were placed in supportive groups with other peers also exhibited positive outcomes through a mechanism of *validation of experience*. Parents struggling with parenting at the same time as recovering from substance abuse (context) were reported to have found the group dynamics and peer relationships fostered with other parents during the COSAP programs to be beneficial. The process of *validation* (mechanism) for these parents can be described as the normalization of experience and sharing of mutual struggles among supportive peers. Further, at least one program evaluation discussed the possibility that the strong bonds formed between participants were a motivator to continue attending sessions (outcome) [38]. It is possible that the creation of supportive peer relationships was a contributing factor to engagement and program commitment, also leading to improvements in parenting skills and child psychosocial outcomes.

Both adults and children appeared to benefit greatly from meeting others and making friends, specifically with people who lived in similar circumstances. For many, this seemed to bring mutual understanding as families’ experiences were normalized and they realized that they were not alone with their struggles. ([29], p. 4)

Thus, according to this demi-regularity, programs that address the social isolation common to substance using families through strategies such as appropriate peer grouping are more likely to trigger mechanisms of validation and trust.

3. (Addiction) Knowledge is power

Programs that specifically emphasized knowledge about addiction and education around the impact that substance abuse has on children and families were found to yield improvements in parenting and child psychosocial outcomes. The following key mechanisms were identified within this demi-regularity: *parental recognition and responsibility* and *children relinquishing responsibility for parental addiction*.

In the Betty Ford program, for example, knowledge was described as “opening the door for them” ([36], p.390) and that simply knowing the truth about their parent’s addiction was helpful. Further, the provision of knowledge (context) allowed children to realize that they were not responsible or at fault for their parent’s addiction (mechanism) [29]. Shame and secrecy (context) are hallmarks of family addiction, according to the family disease model. The mechanism of *relinquishing responsibility* is triggered when children are provided with information about parental addiction that had been previously withheld or downplayed (context). The process of relinquishing responsibility appears to be a key element of the knowledge provision demi-regularity and may set the stage for subsequent improvements in child emotional and behavioural outcomes to occur, either directly through this demi-regularity or through one of the others identified in this review.

Further, parents who attend these programs are also provided with knowledge about how their alcohol or drug use has impacted their children and the family unit. The *parental recognition and responsibility* mechanism is triggered under these circumstances, whereby parents are able to recognize the impact of their behaviours and take responsibility for how it has affected their children. For example, evaluations of the Celebrating Families! program and MPACT program both documented instances where parents gained new understanding of the impact that alcoholism has on the family (mechanism) [39], a realization of not playing the appropriate role as a parent (mechanism) [29] and “the shock that some of the adults conveyed as they began to take in the effects of their lifestyle on their children” ([38], p.18). These programs reported positive outcomes with respect to parenting, such as improved positive parenting and parenting efficacy, by using strategies of knowledge provision to challenge the shame, secrecy and lack of recognition inherent to substance-involved families (context) [38].

### Engaging hard to reach or marginalized families

For certain programs where the participating families were recruited from particularly marginalized populations (e.g. poverty, cultural minority), *engagement* emerged as an important intermediary factor that was necessary in order for outcomes to be achieved. Interestingly, the process of engagement is not explicit within the family prevention model nor the family disease model, perhaps because engagement is assumed to occur once recruitment is established or that engagement is equated with program attendance. As such, engagement did not initially emerge within either candidate program theory. However, as data abstraction progressed, we noted that only those programs classified in this review as aligning with the family prevention model were attuned to this issue. Information on recruitment best-practices exists within family-based intervention literature (e.g. [40]); however, engagement is rarely distinguished from attendance. For the purpose of the present realist review, we conceptualize engagement more broadly than mere program attendance; it refers also to acceptance and uptake of materials. Given the nature of COSAP intervention, engagement is not limited to the client-staff dyad or therapeutic alliance. COSAP programs are group delivery format where clients must engage with each other, the program content and with the program staff. Other realist reviews have identified engagement as an important feature of program success (e.g. [18]). In the present realist review, two instances were identified where successful program engagement yielded positive outcomes: (a) responsiveness to client socio-economic needs and (b) matching to client lived experience. These are discussed below.

- Responsiveness to client socio-economic status (SES) needs. Programs that are responsive to the SES realities and needs of their clients (context) will encourage program engagement (outcome) by fostering a sense of *trust* (mechanism) among families who are typically marginalized (context). For example, SFP and the Safe Haven program both went to extensive lengths to encourage and incentivize participants, such as providing meals, transportation, childcare, basic necessities and vouchers redeemable for family activities. Families participating in these programs were contextually characterized as low income, low education, having unstable housing, child welfare involvement and, unsurprisingly, often mistrustful of service providers. The key mechanism here is the sense of *trust* and acceptance that is developed on the part of the client in response to these staff/program efforts, as evidenced here: “Basic material supports provide a message to needy families that the staff really care about them” ([41], p. 260). In the case of the Safe Haven program, this process was described thusly, as a result of basic necessity provisions:

The Safe Haven staff began to know and understand the unique circumstances of each of the participating families. This seemed to increase staff empathy for the families. The families, in turn, reported to the process evaluator that they felt the staff “cared about them” ([42], p. 46).

In the example above, the program’s responsiveness to client SES needs (context) affected both the staff’s ability to engage with the families and vice versa (outcome), through a process of trust building (mechanism).

b) Matching to client lived experience. Programs that took appropriate steps to match staff and client lived experience of family addiction and/or cultural background (context) were more successful in engaging clients (outcome) by fostering *trust* and personal identification with the program materials (mechanism). This was true of programs such as Safe Haven where extensive efforts to make the program and staff culturally consistent with an African-American worldview led to increases in client acceptance and engagement: “They put it in a way Black people can understand” ([43], p. 233). This program also specifically recruited staff who were themselves also in recovery from substance use (context). Clients noted this shared life experience as being beneficial: “They share of their experiences… this helps” ([43], p. 233). The ability of clients to identify with program content and program staff facilitated *trust* (mechanism) in program and led to increased engagement (outcome). We hypothesize that once engagement is established, other program outcomes will be more easily facilitated via the demi-regularities explored above, creating a series of CMO chains.

Note that our review revealed that cultural adaptation, such as the one described in the Safe Haven program, did not always lead to better outcomes. For example, a number of SFP formats have been culturally adapted for a variety of different ethnic groups in the USA. However, comparisons between generic SFP and culturally adapted versions yielded no improvements in positive outcomes, beyond an increase in retention [44]. Based on the findings above, it is hypothesized that Safe Haven was successful in this regard because the appropriate matching of staff to client lived experience of culture trigged mechanisms of trust and client identification with program materials. Our review did not find evidence of these mechanisms being fired in other culturally adapted programs.

### Alignment with candidate program theories

As part of the analysis process, the four demi-regularities described above were examined with respect to their alignment with the candidate program theories. Programs classified within the family disease model were supported with evidence from the “knowledge” and “supportive peer relationships” demi-regularities. This suggests that the provision of knowledge that is specific to family addiction (context) facilitates children in relinquishing the responsibility for their parent’s addiction (mechanism) and enables parents to recognize and take responsibility for the impact of their addiction on their family (mechanism), leading to improved coping and reduced family stress (outcome). Further, social support provided to families within the context of a supportive peer relationship (context) serves to validate the experiences of families living with addiction (mechanism), leading to improved coping and parenting behaviours (outcomes). The family disease model asserts that defining addiction as a disease is fundamental to the process of relieving oneself from the guilt and responsibility for a family member’s addiction [45]. The “knowledge” demi-regularity supports this theoretical assertion. Additionally, the importance of social support and interpersonal bonding are viewed as essential components of Al-Anon and other support groups that exist within the family disease addiction treatment landscape [45]. While COSAP programs extend beyond the scope of a support group, the “peer relationships” demi-regularity accounts for these findings within family disease model programs.

Programs developed from the family prevention model were evidenced with the “positive parent-child interactions”, “supportive peer relationships” and the “engagement” demi-regularities. This suggests that opportunities for positive parent-child interactions within the context of a safe and supportive environment encourage families to seek joy in spending time together (mechanism) and find hope in the restoration of the family unit (mechanism), ultimately leading to improved family cohesion (outcome). Family prevention theory argues that involving parents in the promotion of healthy child functioning will reduce risk factors and enhance strength and protective factors [10]. The importance of social support in coping with family addiction is also noted in some family prevention literature [46]. The fact that both the “positive parent-child interaction” and “peer support” demi-regularities were found to align with the family prevention model adds evidence for this theory.

Engagement was found to be present within programs originating in family prevention only. Despite the fact that engagement has not previously been noted within COSAP program literature, it has been validated elsewhere. Findings from Jackson and colleagues’ [18] realist review of methadone treatment programs emphasized the importance of client engagement, specifically within the contexts of client-centred treatment, attention to client SES conditions and positive therapeutic relationships. From a broader perspective, other health care fields such as nursing have also emphasized the importance of patient engagement. One particularly useful comparison within the nursing literature is the link between treatment preference, patient engagement and health outcomes [47, 48]. Included in treatment preference is the suitability of the treatment to individual lifestyle [47, 49]. Suitability to personal style could be akin to appropriate matching of client lived experience, as was found in the present study. This alignment was not previously included within the family prevention candidate program theory; as such we conclude this theory should be refined in order to account this finding.

It is notable that two programs included in this review were classified as hybrids, as they drew upon elements common to both candidate program theories. Hybrid programs were evidenced from a combination of all demi-regularities to varying degrees, with the exception of engagement. This is an interesting finding in and of itself but also supports the case for using realist methodology in evaluation inquiry. It would suggest that in practice, program implementation is complex. The MPACT program, for example, was reportedly influenced by the SFP model [38] and was then adapted to the UK context. MPACT maintained policy objectives of improving parent-child communication, parenting skills and child wellbeing [38], which is consistent with SFP and other family prevention model programs. However, our review of the MPACT documents revealed a significant emphasis on understanding the impact of parental addiction on children and families, communicating about addiction and empowering children to take responsibility for their own safety and wellbeing, the latter examples being consistent with family disease model program objectives. The research team concluded that MPACT was best classified as a hybrid, as it appeared to successfully integrate elements from both candidate program theories. The implications of the alignment of our findings within the candidate program theories are further discussed in the next section.

## Discussion

The purpose of this realist review was to explore and expand on two candidate program theories, with the goal of ultimately providing useful guidance for future COSAP program development and implementation. In sum, programs that originate in the family disease model appear to be effective because they facilitate supportive peer relationships and harness the power of knowledge. The key mechanisms responsible for those processes are *trust*, *validation*, *children relinquishing responsibility for parental addiction* and *parental recognition and responsibility*. Family prevention-based programs appear to be effective when they encourage positive parent-child interactions, facilitate supportive peer relationships and are attuned to client engagement. The key mechanisms triggered in those situations are *hopeful enjoyment*, *validation* and *trust*. The interesting case of the hybrid programs highlights that elements from both theoretical orientations can successfully be implemented within a single program. These findings suggest that in practice the differentiation between these two models may be less clear, as some common demi-regularities were noted across candidate program theories (e.g. supportive peer relationships). Given the overlap between theories, the evidence indicates that the CMO configurations uncovered in this review can be situated within a broader and more encompassing COSAP program theory. Previous research has established that COSAP programs can be effective in supporting children and parents living with family addiction (e.g. [13]). This realist review provides evidence for program theory, offering new insights into why and how these programs work. As support was found for both candidate program theories, we conclude that both models have substantial merit. Based on our findings, we propose that a refined program theory emphasize the relationship between the four demi-regularities, such that if the engagement demi-regularity is established first, the other demi-regularities will be more readily generated. When the key mechanism of trust is triggered so as to establish program engagement, uptake of knowledge will be more readily facilitated and likewise the development of peer relationships and opportunities for positive parent-child interactions. This suggests that the contexts identified as integral to engaging hard to reach and marginalized families should be of heightened interest to researchers and practitioners seeking to implement COSAP programming. Further, this review offers implications for future program development and implementation. First, knowledge of the theoretical orientation within which the program model originates can be useful in guiding program implementation. For example, programs developed from a family disease perspective will likely already be attuned to providing knowledge and facilitating supportive peer relationships and, however, may benefit from efforts that encourage positive parent-child interactions. Likewise, programs developed from a family prevention perspective may benefit from additional focus on provision of addiction-specific knowledge in an effort to improve outcomes. Second, the importance of client engagement should be given particular emphasis on the part of program implementers. The interaction of contextual factors (such as client SES, culture and lived experience) with those of the program setting and staffing will impact if and how clients engage and connect with program resources. We noted that the engagement demi-regularity was present in the family prevention programs only. It would be wise for all COSAP programs (including family disease and hybrid models) to pay attention to client engagement and make adjustments where necessary. For example, awareness of client characteristics such as SES or lived experience could inform program delivery through provision of basic needs or appropriate matching of services.

### Limitations of review

A number of limitations are worth noting. Only 7 COSAP programs were found and included in this realist review. While many programs had rich data embedded within the supporting documents, it is entirely possible that other CMOs not reported here might also explain program outcomes. As such we cannot claim that our findings are exhaustive. Second, there were some contextual factors not consistently reported in the evaluations that may have otherwise been relevant. For example, some parents were concurrently enrolled in addiction treatment programs yet no consistent pattern of outcomes could be found. Other parents were described as being “in recovery”, although this was not clearly defined nor were outcomes compared at this level. Level of substance use severity was also inconsistently reported. Child-specific contextual factors were also rarely reported and no discernible patterns of outcomes by age or gender were found across programs. It is possible that had more data been available these contextual factors would have yielded different demi-regularities; however, for the purpose of this review, we concluded that were not enough evidence to confidently make assertions about the impact of those contextual factors. Therefore, while we are confident in the strength of the findings reported, adherence to the CMOs and the demi-regularities identified in this review do not guarantee future program success. Finally, as in any systematic review, the quality and rigour of the evaluations varied considerably. While the realist review methodology is broad in scope and allows for supporting documents to be included to mitigate these factors, there may have been outcomes not sufficiently explored due to methodological constraints of the COSAP program research currently available. This speaks to the need for enhanced evaluation and research of COSAP programs in applied settings.

## Conclusions

This review sheds much needed light on the mechanisms and processes that contribute to COSAP programs’ effectiveness. Yet, it also highlights some significant gaps in the level of evaluation currently being conducted in the family addiction treatment field. Specific attention to intervention fidelity, contextual factors and the increased use of mixed-methods approaches would significantly enhance the rigour and quality of reporting of program evaluations. The inclusion of qualitative studies in the present realist review was highly valuable to our exploration of CMO configurations and demi-regularities. We suspect that had more mixed-methods approaches been reported in the literature, the richness of data uncovered would have been even greater. Moreover, the collecting and reporting of contextual data in evaluation research in this field needs to be more nuanced. Client and facilitator characteristics that are the easiest to measure (e.g. gender, age, level of training) are arguably the least relevant to outcomes [50]. Increased focus on facilitator-client relationships, beliefs about substance abuse treatment and client caseload may be warranted. For example, there is little support in the literature for a main effect of therapist personal addiction history on client outcomes despite the fact that clients often express a desire for such lived experience in treatment [50, 51]. However, it is possible that therapists who are themselves in recovery use different processes to achieve similar outcomes. In the present study, we identified that matching to client lived experience elicited mechanisms of trust and engagement. Further exploration of the process variables associated with facilitator characteristics would improve our understanding of addiction treatment and be consistent with a realist approach to evaluation research. Other examples of more nuanced reporting that could be beneficial include client characteristics such as length of time in recovery, as well as more specific child-related factors. Inclusion of data on these factors could significantly enhance evaluation research and improve the theoretical knowledge base for COSAP interventions.

## Additional files

Additional file 1:
**Description of COSAP Programs and Associated Documents Retained for Review.** This file contains a detailed table of all COSAP programs and their related documents retained for the review. A description of each study and identified contexts, mechanisms and outcomes are included [52–66]. (PDF 88 kb)

## Abbreviations

COA: children of alcoholics
COSAPs: Children of substance abusing parents
CMO: Context-mechanism-outcome
FCP: Family Competence Program
FOF: Focus on Families
MPACT: Moving Parents and Children Together
RAMESES: Realist and Meta-narrative Evidence Synthesis: Evolving Standards
SFP: Strengthening Families Program
