# Supplementary material for: A realist review of family-based interventions for children of substance abusing parents
Source: Syst Rev. 2015 Dec 18;4:177. doi: 10.1186/s13643-015-0158-4 (PMC4683863; doi:10.1186/s13643-015-0158-4)
Supplement: Additional file 1: — Description of COSAP Programs and Associated Documents Retained for Review. This file contains a detailed table of all COSAP programs and their related documents retained for the review. A description of each study and identified contexts, mechanisms and outcomes are included [52–66]. (PDF 88 kb) [file 13643_2015_158_MOESM1_ESM.pdf]

Additional File 1: Table 1

Description of COSAP Programs and Associated Documents Retained for Review

Program Name	Citations	Population and Setting	Evaluation design	Contextual factors	Mechanisms	Selected outcomes reported	Candidate theory alignment
Betty Ford Children's Program	[34, 36]	Predominantly White<br><br>Child age range 7 – 13 yrs<br><br>Mixture of parents in recovery/in treatment/non-addicted partners<br><br>Betty Ford centre in California<br><br>Urban	Pre-post-follow-up design. No comparison group<br><br>Qualitative	Peer grouping<br><br>Knowledge provision	Trust/safety<br><br>Relinquishing responsibility	Reduced child loneliness<br><br>Improved child social skills (younger children and girls only)	Family disease
Celebrating Families!/ Celebrando Familias!	[39, 53, 54, 55, 56]	1) Predominantly non-White<br><br>Mixture of parents in early recovery and other caregivers. Predominantly female<br><br>Child age range 3 – 18 yrs<br><br>Community agencies and treatment centres in San Jose<br><br>Urban<br><br>2) Spanish-speaking	Retrospective pre-post design. No comparison group	Knowledge provision	Recognition and responsibility	Improved family cohesion<br><br>Improved parenting skills	Hybrid

		Hispanic<br><br>Mixture of parents in early recovery and other caregivers<br><br>Child age range 8 – 17 yrs<br><br>Community agencies in California and Oklahoma<br><br>Urban					
Family Competence Program (FCP)	[57, 58]	Spanish (Balearic Islands, Spain)<br><br>Child age range 6 – 14<br><br>Mixture of parents concurrently in drug treatment program and non-addicted partners<br><br>Urban	Pre-post design with comparison group (not randomized)	Opportunities for parent-child bonding	Hopeful enjoyment	Improved family cohesion<br><br>Improved parenting skills<br><br>Improved child behaviours and social skills	Family prevention
Focus on Families (FOF)	[31,32, 33, 59, 60]	Parents predominantly White (At 12-15 yr follow-up, children predominantly identified as non-White)<br><br>Parent concurrently receiving methadone treatment in Seattle<br><br>Child age range 3 – 14 yrs<br><br>Urban	Pre-post design with control group (random assignment)<br><br>Long term follow-up (12-15 yrs post intervention)	SES sensitivity	Hopeful enjoyment (Lack of)	Improved parenting skills<br><br>Reduced parental substance use<br><br>Reduced incidence in child SUD (males only)	Family prevention
Moving	[29, 38, 52,	Predominantly White	Qualitative	Opportunities for	Hopeful	Improved Family	Hybrid

Parents and Children Together (MPACT)	61, 62, 63, 64]	Mixture of parents in treatment/in recovery/still using/non-addicted partners from 13 sites across UK<br><br>Child age range 8 – 17 yrs<br><br>Urban/Rural		parent-child bonding<br><br>Peer grouping<br><br>Knowledge provision	enjoyment<br><br>Trust/safety<br><br>Validation<br><br>Relinquishing responsibility<br><br>Recognition and responsibility	cohesion<br><br>Improved parenting skills<br><br>Improved child behaviours and emotions	
Safe Haven	[30, 42, 43, 65]	African American<br><br>Child age range 6 – 12 yrs<br><br>Mixture of parents concurrently in drug treatment program in Detroit and non-addicted partners<br><br>Urban	Pre-post design. No comparison group	SES sensitivity<br><br>Matching services to lived experience<br><br>Opportunities for parent-child bonding	Trust<br><br>Hopeful enjoyment	Improved family cohesion<br><br>Improved child behaviours (high drug using families only)<br><br>Reduced parental substance use	Family prevention
Strengthening Families Program (SFP)[a]	[11, 27, 35, 37, 41, 44, 66]	1) Parents concurrently in outpatient drug treatment (methadone or other outpatient) in Salt Lake City, Utah<br><br>Child age ranges 6 – 12 yrs<br><br>Urban<br><br>2) African American<br><br>Mixture of mothers in drug treatment in rural Alabama	Pre-post design with control group (random assignment)<br><br>Pre-post-follow up design with comparison group (not randomized)<br><br>Quasi-experimental retrospective pre-post design with post-hoc	SES sensitivity<br><br>Opportunities for parent-child bonding<br><br>Peer grouping	Trust/safety<br><br>Hopeful enjoyment<br><br>Validation	Improved family cohesion<br><br>Improved child behaviours and emotions<br><br>Improved parenting skills	Family prevention

	and not in treatment<br><br>Rural<br><br>3) Predominantly non-White (Asian, Pacific Islander, American Indian, Hispanic)<br><br>Mixture of parents in treatment/recovery/non-addicted partners<br><br>Child age range 6 –13 yrs<br><br>4) Multi-ethnic<br><br>Mixture of parents currently in treatment, not in treatment, in recovery in New Jersey<br><br>Child age ranges: 3-16 yrs<br><br>Urban<br><br>5) Predominantly White<br><br>Mixture of alcoholic parents and non-alcohol spouses in Ontario, Canada<br><br>Child age ranges: 9 – 12 yrs<br><br>Urban	comparison groups

[a] SFP was originally designed for COSAPs; however subsequent program implementations have been revised to include at-risk youth whose parents are not substance users. Only documents/evaluations specific to COSAPs were retained for this review.
SUD = substance use disorder. SES = socio-economic status
